# Current Understanding of Marginal Grafts in Liver Transplantation

**DOI:** 10.14336/AD.2024.0214

**Published:** 2024-02-14

**Authors:** Ze Xiang, Jiarui Li, Huixuan Zeng, Xiaonan Xiang, Fengqiang Gao, Kai Wang, Xuyong Wei, Shusen Zheng, Xiao Xu

**Affiliations:** ^1^Department of Hepatobiliary and Pancreatic Surgery, Hangzhou First People's Hospital, Hangzhou 310006, China.; ^2^Zhejiang University School of Medicine, Hangzhou 310058, China.; ^3^Department of Surgery, University of Cambridge and NIHR Cambridge Biomedical Research Centre, Cambridge, Cambridgeshire, UK.; ^4^Shulan (Hangzhou) Hospital, Zhejiang Shuren University School of Medicine, Hangzhou 310022, China.; ^5^NHC Key Laboratory of Combined Multi-organ Transplantation, Hangzhou 310003, China.

**Keywords:** marginal grafts, liver transplantation (LT), usability evaluation, risk factors, improved methods

## Abstract

End-stage liver disease (ESLD), stemming from a spectrum of chronic liver pathologies including chronic liver failure, acute cirrhosis decompensation and hepatocellular carcinoma, imposes a significant global healthcare burden. Liver transplantation (LT) remains the only treatment for ESLD. However, the escalating mortality on transplant waitlists has prompted the utilization of marginal liver grafts in LT procedures. These grafts primarily encompass elderly livers, steatotic livers, livers from donation after circulatory death, split livers and those infected with the hepatitis virus. While the expansion of the donor pool offers promise, it also introduces concomitant risks. These encompass graft failure, biliary and cardiovascular complications, the recurrence of liver disease and reduced patient and graft survival. Consequently, various established strategies, ranging from improved donor-recipient matching to surgical interventions, have emerged to mitigate these risks. This article undertakes a comprehensive assessment of the current landscape, evaluating the viability of diverse marginal liver grafts. Additionally, it synthesizes approaches aimed at enhancing the quality of such marginal liver grafts. The overarching objective is to augment the donor pool and ameliorate the risk factors associated with the shortage of liver grafts.

## 1. Introduction

The concept of end-stage liver disease (ESLD) was introduced in the 1980s and refers to the advanced stages of liver disease stemming from various etiologies of chronic liver damage [1]. This condition is typified by the liver's incapacity to fulfill the body's physiological needs [2]. Its scope includes the end-stage of various chronic liver diseases, including acute-on-chronic liver failure, acute decompensation of cirrhosis, chronic liver failure and hepatocellular carcinoma (HCC) [3, 4]. Liver transplantation (LT) remains the only effective treatment for ESLD. After a boom in the 1980s, especially with the widespread use of deceased donor organ procurement, the LT industry grew quickly, contributing to a notable increase in the quantity and caliber of transplants performed [5].

Due to the recent shortage of liver donors and increased mortality on the waiting list, there is an increasing trend to transplant elderly livers, steatotic livers, donation after circulatory death (DCD) livers, split livers and hepatitis virus-positive livers. However, the utilization of these suboptimal grafts can be associated with poor outcomes, including allograft rejection, graft loss, vascular complications, biliary complications, recurrent liver diseases and shorter survival. As a consequence, these grafts are often designated as "marginal liver grafts" [6, 7]. The proportion of marginal livers used in LT is increasing every year [8]. Nowadays, several marginal livers have achieved good outcomes, which is attributed to advances in surgical techniques, the development of immunosuppressive agents and improvements in related medical technologies. Nevertheless, efforts to improve the quality of marginal liver to increase the pool of viable livers are one of the principal issues in the field of LT.

This article reviewed the prevailing circumstances and systematically evaluated the applicability of various marginal liver grafts, mainly including elderly livers, steatotic livers, DCD livers, split livers and hepatitis virus-positive livers. Moreover, we also summarized methods to improve the quality of marginal liver grafts, aiming to expand the donor pool and alleviate risk factors associated with liver graft shortages.

## 2. Elderly liver grafts

### 2.1 Definition

In recent years, the average age of the general population has continued to increase [9], leading to a rise in the number of elderly liver donors [10, 11]. From 1990 to 2015, the average age of donors reported in European transplantation arenas exhibited an elevation from 35 to 54 years old, and the use of liver grafts from donors >60 has been studied and applied more widely [12]. Elderly liver donors usually manifest shared characteristics, including a higher proportion of female donors, a diminished glomerular filtration rate (GFR) and an elevated prevalence of concurrent diseases, notably hypertension [13] and diabetes mellitus [14]. The main causes of death among elderly liver donors are cerebrovascular accidents, followed by trauma (81% vs 15%) [14]. Consequently, older donors tend to have shorter stays in the intensive care unit (ICU), fewer hemodynamic instabilities or cardiac arrests and lower laboratory markers such as serum sodium and transaminase levels [14].

Nowadays, the application of elderly liver grafts is limited by concerns about poor outcomes and high rejection rates associated with elderly liver grafts [15]. Considering the constantly changing donor environment and the goal of lowering mortality on the waiting list, there is an urgent need to enhance the utilization of elderly liver grafts.

**Table 1:** Postoperative complications after using elderly liver grafts for transplantation.

Complications	Research subject	Outcomes	Ref.
**Allograft rejection, graft loss and recurrent malignancy**	Liver transplant recipients of elderly liver grafts (n = 78); Matched controls (n =78)	Elderly liver grafts were associated with early rejection but not with graft failure and malignancy.	[13]
**Allograft rejection**	Living-donor liver transplantation cases of different donor ages (elderly, n = 36; non-elderly, n = 434)	Donor age was not significantly associated with 6-month graft survival.	[16]
**Graft loss**	Liver transplant recipients of different donor ages (elderly, n = 167; non-elderly, n = 187)	Donor age was not a predictor of graft loss.	[17]
**Graft loss**	Liver transplant recipients of different donor ages (n = 63,628)	The use of elderly liver grafts had different effects on recipients of different ages.	[9]
**Graft loss and vascular complications**	Liver transplant recipients with hepatocellular carcinoma (n = 4,971)	No significant difference in graft loss and vascular complications between elderly and non-elderly grafts.	[18]
**Vascular complications and biliary complications**	Liver transplant recipients (n = 4,376)	No statistically significant difference in biliary and vascular complications.	[19]
**Biliary complications**	Orthotopic liver transplantation cases (n = 1,843)	Donor age was significant risk factors for the development of ischemic-type biliary lesions.	[21]
**Biliary complications**	Orthotopic liver transplantation cases (n = 749)	No association between donor age and incidence of non-anastomotic biliary strictures.	[22]
**Biliary complications**	Liver transplant recipients of different donor ages (elderly, n = 68; non-elderly, n = 136)	No difference in the incidence of biliary complications between these two groups.	[24]
**Recurrent HCC**	Liver transplant recipients of elderly liver grafts (n = 25)	Elderly liver grafts may be linked to recurrent HCC.	[25]

### 2.2 Usability evaluation

#### 2.2.1 Postoperative complications (Table 1)

##### 2.2.1.1 Allograft rejection and graft loss

Although the rate of graft loss has decreased in recent years due to improved transplantation techniques, allograft rejection still stands out as a major complication after LT. Schneider *et al.* illuminated the role of elderly liver grafts as a substantial determinant of rejection since the adoption of aged liver grafts resulted in a significant rise in early rejection [13]. Similarly, Kadohisa *et al.* also found that recipients of elderly liver grafts showed higher transaminase peaks after LT, irrespective of their age; this elevation in transaminase levels is often correlated with postoperative rejection [16].

However, the risk of graft loss using elderly liver grafts after LT remains a controversial issue. Bittermann *et al.* reported that the risk of graft loss rose with increasing donor age in younger recipients (referred to as <40 years old) [9]. However, Schneider *et al.* obtained a different conclusion that advanced donor age was not a significant factor leading to graft loss [13]. Roullet *et al.* demonstrated that donor age was not the predictor of graft loss [17]. Similarly, in a nationwide retrospective cohort study, Hu *et al.* found no significant difference between HCC patients receiving elderly and younger liver grafts in terms of graft loss after eliminating the influence of high Model for End-Stage Liver Disease (MELD) scores [18].

##### 2.2.1.2 Vascular complications

Advanced donor age was not a predictor of vascular complications in multiple data sets. A meta-analysis comparing the result difference of adult patients undergoing LT using grafts from <70-year-old or >70-year-old donors found that liver grafts from selected >70-year-old donors did not pose added organ-specific risks, thus having comparable transplantation outcomes [19]. A similar study also showed that for appropriately selected matched recipients, accepting elderly donor livers will not increase the risks of vascular complications [18].

##### 2.2.1.3 Biliary complications

Biliary complications represent one of the leading causes of mortality and morbidity after LT [20]. Haydenhain *et al.* retrospectively studied 1843 patients who underwent orthotopic liver transplantation (OLT) and found that donor age was associated with a significant effect on the development of ischemic-type biliary lesions [21]. However, another study encompassing 749 patients showed no association between donor age and incidence of non-anastomotic biliary strictures (NAS) [22]. Mils *et al.* also indicated no difference in the incidence of biliary tract complications between receptors of more or less than 70-year-old grafts [23]. The results of the study by Westercamp *et al.* similarly indicated that transplantation of livers from elderly donors (≥65 years old) was not associated with a higher incidence of biliary complications when the cold ischemia time (CIT) was kept short [24].

##### 2.2.1.4 Recurrent liver diseases

Recurrent malignancies, represented by HCC, can also pose a threat to the health of patients receiving LT. In this regard, Kim *et al.* evaluated the survival and incidence of the main post-transplant complications in 25 patients who received livers from donors older than 70 years old and found that the causes of graft losses for the older donor livers included recurrent HCC [25]. Likewise, Schneider *et al.* embarked on a retrospective analysis of data encompassing LT recipients aged 80 years and older, who had undergone the procedure using liver grafts from two distinct centers in the US during the period spanning 2000 to 2011. Their findings demonstrated that liver transplantation from donors aged ≥80 years did not translate into increased risk of post-transplant cardiovascular diseases, hypertension, vascular complications, or malignancies in elderly recipients [13].

#### 2.2.2 Patient and graft survival

Multiple studies have shown the impact of elderly liver grafts on post-transplant mortality, a phenomenon that exhibits variability in the age of recipients, particularly among younger recipients [9, 26, 27]. LT from donors aged 60 or older has great transplant outcomes, with no significant difference in 1-year and 5-year survival rates compared with patients receiving old and normal liver grafts [28-30]. Moreover, a study by Haugen *et al.* designed a nationwide study of 1,311 patients who accepted elderly liver grafts and 23,709 patients who declined such grafts. This exploration revealed a notable survival advantage for those who accept elderly liver grafts, where heightened MELD scores correlated with decreased mortality rates [31]. It is noteworthy that both older and younger candidates gain survival benefits from receiving the old grafts. Recipients and donors should carefully weigh the consequences of rejecting the old graft offer, as a quarter of candidates died following declining the offer. These findings will guide patients in their future clinical decisions.

### 2.3 Expanding the liver donor pool

#### 2.3.1 Donor-recipient matching

It is essential to evaluate outcomes and minimize risks when using elderly liver grafts. Optimized donor-recipient matching is key to achieving good results [32, 33]. The MELD score is the main model for end-stage liver disease [34]. In this context, Haugen *et al.* evaluated the effectiveness of "preferred recipients" (MELD score 6-40) by comparing recipients of old donors (≥70 years) with recipients of average liver donors (ALDs, 40-69 years) and ideal liver donors (ILDs, 18-39 years) based on an analysis of 38,891 LT patients from 2006 to 2013. This retrospective cohort study found that preferred recipients for old donors had similar graft loss rates to those for ALD and ILD. These results indicate that matching older donor grafts with better MELD scores in recipients does not increase graft loss or post-transplant mortality associated with donor age [35]. Considering the impact of donor and recipient age on LT outcomes, new scoring systems have emerged in recent years, including the donor MELD (D-MELD) [36] and the donor-recipient MELD (DR-MELD) [14]. Employing the DR-MELD framework, Caso-Maestro *et al.* demonstrated that patients with a DR-MELD score ≤75000 exhibited a 5-year graft survival rate exceeding 70%, while patients with a DR-MELD score ≥75000 had a 5-year graft survival rate below 50% [14], providing some guidance for future LT donor-recipient matching.

Numerous studies have shown that the risk of liver graft loss from elderly donors remains independent of recipient age, providing insight into the matching of elderly liver donors with recipients [27, 9]. However, for high-risk recipients, careful evaluation and selection of appropriate donors are necessary to avoid adverse prognostic issues. Careful evaluation and selection, which involves avoiding factors such as hypernatremia, prolonged ICU stay, liver enzyme alterations and cardiac arrest, can render the usage of liver grafts from donors aged over 80 years in LT [37, 38]. Therefore, age alone should not be an absolute contraindication for LT; rather, risk assessment should be stratified based on factors like MELD, facilitating the discerning selection of elderly liver donors for recipients. This approach reduces the risk of using such donors and expands the donor pool safely.

#### 2.3.2 Machine perfusion

Furthermore, the suboptimal performance of elderly liver grafts predominantly stems from their vulnerability to ischemia-reperfusion injury [39]. In a comprehensive exploration encompassing 3,104 elderly LT donors (aged >70 years), Halazun *et al.* identified the only donor risk factor as CIT exceeding 8 hours [40]. In clinical practice, Pratschke *et al.* recommended avoiding pairing advanced-age liver donors with high-risk factors such as high urgency, hepatitis C virus (HCV) infection, elevated MELD score and prolonged CIT [41]. Gao *et al.* proposed that CIT reduction emerges as a major factor in expanding the pool of liver donors, necessitating the evolution of ex vivo liver perfusion systems [30].

Collectively, a large body of evidence suggests that the outcomes of LT employing elderly donors are comparable with those involving younger donors after the optimization of donor-recipient matching. The risks of graft loss and mortality exhibit no substantial correlation with donor age, and elderly donor livers do not increase the incidence of complications such as biliary or vascular complications. There are still persistent challenges, including a longer recovery period and poor wound healing. The emergence of ex-vivo liver perfusion systems introduces the potential to diminish the risk of cold ischemia injury in elderly donor livers, providing favorable conditions for selective expansion of the donor pool. Given the persistent shortage of organs and the increasing average age of liver donors, it is necessary to advocate for the increase in the utilization of elderly donor livers.

## 3. Steatotic liver grafts

### 3.1 Definition

With the increasing incidence of obesity and non-alcoholic fatty liver disease (NAFLD), the global prevalence of liver steatosis has risen among organ donors [42]. Currently, the main methods employed to evaluate hepatic steatosis include gross visual recognition, imaging analysis [43], controlled attenuation parameter (CAP) [44], and biopsies [45]. Steatotic donor livers (SDLs), defined as livers with severe steatosis (≥30% fat content), have emerged as a new source for expanding the donor pool. Hepatic steatosis (HS), characterized by the infiltration of fat in tissue slices, is quantitatively defined as <30%, 30-60%, or >60% fat content and qualitatively as mild, moderate, or severe [46]. Traditionally, steatotic livers have been categorized into two principal histological types: macrovesicular and microvesicular steatosis. Macrovesicular steatosis is identified by a single large vacuole that exceeds the nucleus in size, often displacing the nucleus toward the cell membrane. On the contrary, microvesicular steatosis is marked by small lipid vesicles without nuclear displacement, resulting in a foamy aspect of the cytoplasm [46].

**Figure 1.**
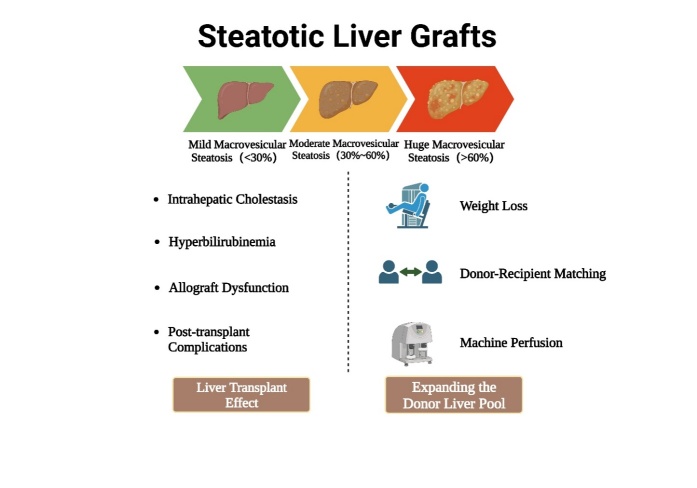
Steatotic liver grafts.

Significantly steatotic livers for transplantation can be identified by their yellow discoloration (especially upon sectioning), round edge, absence of scratch marks and greasy and firm texture [47]. However, gross visual appearance does not provide morphological data on the type of fat infiltration (comparison between macro- and microsteatosis) [48]. Additionally, it is susceptible to subjectivity, rendering its clinical utility a topic of ongoing discourse. Given the marginal status of numerous deceased donors, surgical teams often request biopsies to evaluate the severity of steatotic liver [49]. Currently, the evaluation of fresh frozen liver biopsies maintains its position as the gold standard for appraising the suitability of liver transplants [43, 44]. Broering *et al.* validated the CAP, a qualitative and quantitative technique that measures hepatic fat attenuation at a frequency of 3.5 MHz. This technique was found feasible for the in vivo assessment of hepatic steatosis in living liver donors [44]. It is worth noting that the CAP threshold varies based on the underlying etiology and can proficiently discern substantial hepatic steatosis in patients afflicted with viral hepatitis, albeit potentially exhibiting limitations in characterizing steatosis in individuals with NAFLD [50] (Fig. 1).

### 3.2 Usability evaluation

Historically, steatotic liver grafts were often discarded due to elevated mortality and graft loss rates [51]. In the short term, significant hepatic steatosis has emerged as a prevalent risk factor for primary non-function (PNF), graft dysfunction and biliary complications [49, 52, 53]. However, in the contemporary landscape of LT, high-fat grafts with massive hepatic steatosis levels of 30% or beyond, are experiencing heightened utilization, with transplant outcomes equivalent to those of non-steatotic livers, when other donor risk factors are minimized.

#### 3.2.1 Postoperative Complications

##### 3.2.1.1 Allograft rejection and graft loss

Donor liver implantation with pronounced fat infiltration (>60%) has been reported to be generally associated with early liver dysfunction and an increased incidence of PNF after LT. A comprehensive biopsy of 124 donor livers conducted pre-transplantation unveiled a striking correlation, with 7 out of 8 livers with PNF exhibiting concurrent severe fat infiltration [54]. Marsman *et al.* evaluated the outcome of LT with donors containing up to 30% fat. The use of a donor liver with fat infiltration was shown to be significantly associated with a reduction in both 4-month graft survival and 2-year patient survival when graft and patient survival were compared with controls. PNF and primary dysfunction are the predominant causes of graft loss and mortality [55].

##### 3.2.1.2 Vascular complications

To date, the predominant consensus suggests that the prevalence of vascular issues has not demonstrated a substantial correlation with the extent of steatosis. For instance, Ma *et al.* conducted a retrospective analysis of clinical data encompassing 199 adult LT donors and recipients. Within the cohort of elderly donors (aged ≥60 years), they further divided the group into those with lower fat infiltration (<20%) and higher fat infiltration (≥20%) based on the degree of donor liver fat content. Their study found that there was no significant difference in the incidence of vascular complications [56]. Similarly, other studies have also found no appreciable difference in the incidence of vascular problems between groups with lower and higher fat infiltration [57].

##### 3.2.1.3 Biliary complications

Growing evidence from both clinical and experimental studies showed that steatosis in LT exerts a detrimental impact on the manifestation of post-transplant biliary complications. 117 consecutive deceased donor LTs were retrospectively analyzed for the development of biliary complications and univariate analysis indicated that macrovacuolar steatosis of the graft >25% was a significant predictor of biliary complications [58]. Perhaps, a poorly functioning sinusoidal microcirculation could be responsible for ischemic damage to the bile duct, leading to an increased risk of biliary strictures. However, this initial hypothesis needs further investigation [59].

##### 3.2.1.4 Recurrent liver diseases

Steatosis within the liver graft has emerged as a negative prognostic factor for HCV recurrence [60]. However, the precise mechanisms by which graft steatosis influences the recurrence of HCV following transplantation remain unclear. In contrast to earlier findings, a recent study found no evidence that steatotic grafts accelerated the progression of fibrosis in HCV patients [61]. Apart from HCV, the ramifications of graft steatosis extend to other spheres. Orci *et al.* found that in both the univariable and multivariable analyses, only severe graft steatosis (>60%) was independently associated with an increased risk of recurrence of HCC [62].

#### 3.2.2 Patient and graft survival

For LT involving steatotic grafts, it is crucial to distinguish between different types of hepatic steatosis. On this basis, Croome *et al.* compared the clinical outcomes of LT recipients with moderate macrovesicular steatosis (30%-60%) from donors to those with mild macrovesicular steatosis (10%-29%) or without steatosis between 2000 and 2017 using propensity score matching. The degree of steatosis was found to be correlated with postreperfusion syndrome (PRS), early allograft dysfunction (EAD), the need for continuous renal replacement therapy and the proportion of patients who recovered to OR within 30 days post-transplant. However, no statistically significant variance appeared in terms of long-term patient and graft survival [63]. In other words, if patients can overcome the initial perioperative risks associated with using these livers, long-term graft survival can reach a similar level to that of normal livers. A separate study involving 67 recipients of living donor liver transplant (LDLT) unveiled a significant correlation between elevated macrovesicular steatosis before surgery and increased incidence of intrahepatic bile stasis and hyperbilirubinemia during the post-transplant regeneration phase. Moreover, heightened macrovesicular steatosis before surgery was linked to an increased occurrence of PNF [64]. Therefore, improving the utilization of SDLs may be a reasonable strategy to expand the donor pool.

In summary, as the field of LT continues to advance, patient survival and graft outcomes for steatotic LT have improved. When a steatotic graft becomes the only viable option and there is an urgent demand for LT, a mildly large steatotic donor liver can perform at a level comparable to a normal donor liver and a moderately steatotic donor liver should be taken into consideration when focusing on long-term survival rates. Furthermore, the use of grafts with severe steatosis may save the lives of many patients who are dying on the waiting list.

### 3.3 Expanding the liver donor pool

#### 3.3.1 Weight loss

The risk of steatosis for LT mainly stems from the degree of macroalveolar steatosis. Addressing this facet can enhance the efficacy of marginal donor liver utilization and the overall success of transplantation endeavors. Perkins *et al.* showed that weight loss can yield a reduction in macroalveolar steatosis. This intervention proved to be effective in reversing the degenerative course of steatosis. The levels of peak total bilirubin, postoperative prothrombin time and peak alanine aminotransferase showed no difference between the treated and control groups by the end of the study cycle and the rate of post-LDLT complications decreased [65]. Likewise, similar studies present the same results [66-68]. A meta-analysis indicated that a significant decrease in BMI brought a reduction in steatosis with a mean difference of -21.22 [69]. Therefore, the pool of living donors can be expanded through lifestyle interventions featuring well-structured weight loss programs and judicious dietary supplementation. It is also important to note that weight loss in any modality can achieve the same effectiveness as pharmacotherapy, but potential side effects, including hepatotoxicity, should be considered [53].

#### 3.3.2 Donor-recipient matching

To minimize the potential risks associated with steatotic liver donors, donor-recipient matching seems to be a necessity. Jackson *et al.* introduced a noteworthy paradigm termed the "preferred recipients" concept, which strategically identified and categorized recipients who lack factors exacerbating the impact of donor steatosis on mortality and graft loss. These preferred recipients encompassed individuals with MELD scores ranging from 15 to 34, free of primary biliary cirrhosis and not on pre-transplant life support. These candidates with relatively good clinical status but with a high demand for liver grafts may not receive transplantation before death while on the waiting list [59]. Cox regression analyses unveiled that selecting preferred recipients of steatotic livers did not lead to elevated risks of death or graft loss in transplantation using steatotic liver grafts. Conversely, steatotic liver in non-preferred recipients was associated with heightened risks of death and graft loss compared to non-steatotic livers [51]. Therefore, the assessment of LT recipients is crucial for the outcome of steatosis donor LT, and recipients with low MELD scores and good physical condition should be given priority to be selected.

Matching the degree of steatosis and BMI has also been shown to be effective in improving the outcome of LT with macrosteatotic livers. Northup *et al.* found that both macrosteatotic livers and high-BMI recipients increased the risk of death one year after transplantation. Notably, the combination of macrosteatotic livers and high-BMI recipients showed the most unfavorable outcomes, while the combination of low-fat livers and normal-BMI recipients yielded the most favorable result [49]. Consequently, low-BMI recipients should be given priority when considering macrosteatotic livers for transplantation. This highlights the importance of recipient evaluation in the context of steatotic LT.

The paradigm of integrating the risk profile of donors and recipients into an organ allocation may enhance the utilization of donated organs and improve transplant outcomes. The inclusion of donor-recipient matching as part of liver organ allocation research in the future can further improve LT outcomes and minimize organ wastage.

#### 3.3.3 Machine perfusion

Machine perfusion (MP) systems, including pre-harvest in situ normothermic oxygenated perfusion (NMP), in situ normothermic MP or hypothermic oxygenated perfusion (HOPE) after organ retrieval and transfer to the transplant center, have been developed with de-fatting approaches. The utilization of MP not only curtails ischemic time but also mitigates the risk associated with steatotic liver transplantation [46]. Notably, NMP exhibits the potential to modulate lipid metabolism under certain conditions [70]. In the future, an exploration of supply-demand alignment as part of liver organ allocation can further improve LT outcomes while minimizing organ wastage.

HOPE demonstrates the capability to prevent reperfusion injury and enhance post-transplant liver function. Notably, the historical clinical experience accentuates that cold-preserved steatotic liver transplants evoke significant reperfusion injury following OLT in contrast to non-steatotic LTs. The integration of HOPE treatment after cold preservation, while incapable of altering the inherent degree of steatotic degeneration, manages to lower oxidative stress levels, mitigate nuclear damage, suppress the activation of Kupffer and endothelial cells, curtail fibrosis within the initial week post-surgery and substantially attenuate reperfusion injury ensuing LT [71].

However, it should be noted that the steatotic liver is more susceptible to ischemia-reperfusion injury (IRI). Due to impaired hepatic microcirculation, steatotic livers may have reduced tolerance to IRI [72, 73]. Furthermore, the amplification of mitochondrial oxidative damage and lactate accumulation, engendered by an inefficient anaerobic metabolism during hepatic IRI, emerges as a principal mechanism leading to reperfusion injury [72].

In summary, MP can serve as both pharmacological and non-pharmacological interventions to reduce CIT time, particularly in de-fatting approaches, to improve the quality of liver grafts from steatotic donors and expand the donor pool.

## 4. DCD liver grafts

### 4.1 Definition

DCD involves the procurement of organs for transplantation after confirmation of death based on circulatory criteria [74]. This practice extends to LT, encompassing two distinct pathways: controlled DCD (cDCD) and uncontrolled DCD (uDCD), although experience with uDCD liver donation remains limited [75]. Overall, compared to donors after brain death (DBD), DCD donors have longer warm ischemia time (WIT), more organ damage and often have worse outcomes, marked by elevated rates of mortality, graft loss and biliary complications. This historical scenario has led to a higher rate of discarded DCD livers [76]. Additionally, with the increasing prevalence of complications such as diabetes and obesity, as well as an increase in DCD donations, it is expected that the discard rate for DCD livers will continue to rise [77].

The landscape, however, is poised for change. The relentless demand for LT surpasses the supply of "suitable" organs [78]. Modifications in the donor pool, US organ allocation policies and clinical protocols could influence the risk of complications post-DCD LT [77, 79]. Furthermore, current research has demonstrated that DCD LT offers a lower risk of mortality and provides higher quality-adjusted life years compared to waiting for DBD LT, sparking renewed attention from healthcare professionals [76]. In regions where the MELD score is elevated, organs from DBD donors typically gravitate towards patients with higher MELD scores. Consequently, for patients with liver cancer and/or lower MELD scores, DCD donors and living donor (LD) LT are viable alternatives. Although the proportion of Chinese citizens voluntarily donating organs after cardiac death has declined, more recipients are considering using grafts from DCD donors for LT, seeking to counteract organ scarcity and mitigate waiting list mortality [80].

**Figure 2.**
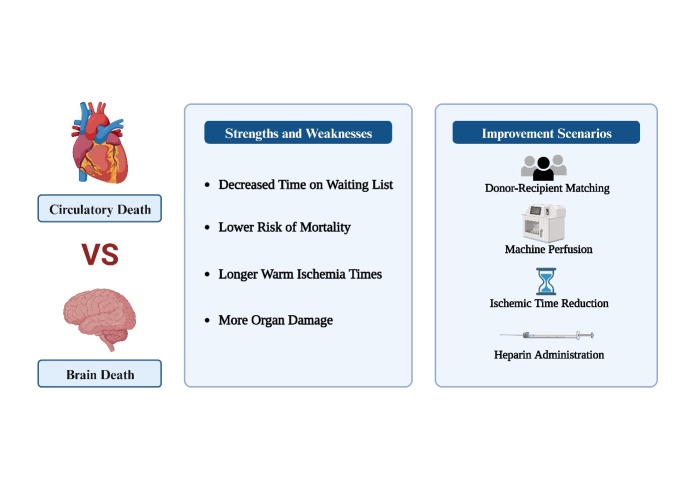
Clinical performance and improve methods of donation after circulatory death livers.

Moreover, the adoption of cDCD and uDCD strategies poses a plethora of ethical, legal and moral challenges, including the definition of refractory cardiac arrest, the time limit for organ ischemia, the required time and type of consent, determination of death and potential conflicts of interest between life-saving interventions and preservation of organ function through extracorporeal membrane oxygenation [81, 82] (Fig. 2).

### 4.2 Usability evaluation

Current research mainly focuses on comparing the outcomes of DCD LT with those of DBD LT. This encompasses a comprehensive examination of both short- and long-term patient and graft survival rates, postoperative graft failure, as well as a thorough scrutiny of associated biliary and vascular complications. Moreover, considerable discourse revolves around factors that correlate with the efficacy of DCD LT, particularly concerning distinct categories of DCD liver donors.

#### 4.2.1 Postoperative complications

Postoperative complications pose a paramount concern in the context of DCD LT. Among these, prevalent issues encompass PNF, EAD, ischemic cholangiopathy (IC) and hepatic arterial complications (HACs). The most severe complication post-OLT pertains to the allograft’s primary nonfunction, often stemming from uncontrolled circulatory death of the liver (uDCD) [83]. Notably, DCD LT is marked by a significantly elevated risk of biliary complications and anastomotic/non-anastomotic leak and stricture (AS/NAS) compared to DBD [84]. An in-depth analysis of graft failures indicates that even three years post-transplantation, the DCD group continues to exhibit a significantly higher rate of biliary complications compared to the DBD cohort, underscoring the persistent nature of this challenge [85].

##### 4.2.1.1 Allograft rejection and graft loss

Historically, DCD has consistently exhibited elevated rates of graft failure in comparison to DBD. To elucidate the distinct independent risk factors contributing to graft failure post-OLT, a thorough analysis was conducted on 81 patients who underwent OLT employing DCD grafts from 1994 to 2010. According to the findings, graft failure occurred in 14 patients (17%), with a median survival of 5.4 months [86]. In another comprehensive study, Meier *et al.* collected and examined data from a UNOS single-center cohort, encompassing the survival outcomes of DCD, DBD and LDLT patients/grafts from 1989 to 2019. Their investigation unveiled that the risk of graft loss in DCD-LT was 1.7-fold and 1.3-fold greater when contrasted with DBD-LT and LDLT, respectively [84]. Fortunately, over time, the risk associated with mortality in DCD LTs witnessed a gradual decline across all time points, accompanied by a commensurate reduction in the risk of graft loss [76].

##### 4.2.1.2 Vascular complications

An elevated susceptibility to vascular complications is also associated with the utilization of DCD. Pine *et al.* undertook a comparative study involving 39 DCD recipients with a case-matched group of DBD recipients. Within the DCD group, 6 instances of vascular complications emerged, comprising 5 cases of hepatic artery stenosis (HAS) and 1 case of hepatic artery thrombosis (HAT). Notably, the incidence of HAS was significantly higher in the DCD group [87]. However, a differing perspective was presented by Vivalda *et al.* who reviewed potentially relevant English-language articles published from 2012 to 2017. Their findings indicated that DCD liver grafts did not exhibit an escalated risk of vascular stenosis in comparison to DBD [88]. Moreover, many studies reached the same conclusion [89, 90]. Therefore, it remains to be determined if a marginally elevated rate of vascular complications can be expected when employing DCD donors with advanced age.

##### 4.2.1.3 Biliary complications

The post-transplant biliary complications associated with DCD transplantation have garnered significant attention within the literature. Hong *et al.* reported that among the 81 patients, biliary complications occurred in 24 cases (29%), including 8 instances of IC and 16 cases of anastomotic leaks or strictures [86]. A meta-analysis conducted by O’Neill *et al.* showed an overall incidence of biliary complications at 26% for DCD liver grafts, compared to 16% in DBD grafts [91]. A single-center retrospective study also found that IC rates were significantly higher among DCD recipients compared to DBD patients [92].

##### 4.2.1.4 Recurrent liver diseases

The rates of post-transplant tumor recurrence vary between centers due to the diverse selection criteria applied. According to Orci *et al.*, the transplantation involving DCD did not exert a significant impact on HCC recurrence, nor did the duration of CIT. However, individuals with prolonged WIT showed an increased HCC recurrence risk [62]. Yet, the influence of DCD utilization on tumor recurrence post-transplantation for HCC remains a controversial topic, although several studies have indicated no substantial difference between DCD and DBD [8, 93].

##### 4.2.2 Patient and graft survival

The number of DCD LTs is on a steady rise and the data reflect continuous improvements in results. Considering the potential of thrombolytic therapy to reduce vascular and biliary complications, Bohorquez *et al.* classified 100 consecutive DCD livers into two groups: thrombolytic therapy (late DCD) and historical DCD (early DCD) groups, both of which were then compared with DBD group [94]. The findings demonstrated that the 1- and 3-year transplant survival rates for late DCD LT recipients surpassed those of early DCD LT recipients. Moreover, through the implementation of a protocol involving thrombolytic therapy, DCD LT achieved comparable patient and graft survival to DBD LT. Schlegel *et al.* studied the use of DCD at >60 years of age and found interesting results [95]. Incidences of vascular, biliary and overall complications were relatively low, with the median composite complication index proving independent of donor age. This suggests that when other risk factors are minimized, elderly DCD donors can be effectively employed in liver transplantation with favorable long-term outcomes. While numerous studies have been made on the feasibility of utilizing adult donors, data regarding the use of pediatric DCD donors remains limited and requires further advancement [78].

Despite the encouraging progress in outcomes, IC remains the Achilles heel of DCD LT. Concurrently, the concept of functional donor WIT has emerged due to unique circumstances in the DCD procurement process, including hemodynamics, forced waiting period, and time intervals from aortic incision to intubation and cross-clamping. These variables can yield varying effects on the outcome of LT [96, 97]. Researchers are presently seeking strategies to tackle these issues and broaden the list of potential liver donors.

#### 4.3 Expanding the liver donor pool (Table 2)

While notable strides have been achieved in enhancing the outcomes of DCD LT recipients, DCD livers continue to face a higher likelihood of being discarded compared to DBD livers. This discrepancy has become more pronounced over time. Data reveals that over the past decade, the rate of growth in DCD LTs procedures conducted in the United States has surpassed that of DBD LTs [98]. Remarkably, DCD livers remain underutilized, holding significant potential to augment the available donor pool and thereby alleviating the persistent scarcity of organs.

**Table 2:** Methods to expand the liver donor pool in steatotic and DCD liver transplantation.

Methods	Type of liver graft	Research subject	Outcomes	Ref.
**Weight loss**	Steatotic liver graft	Living-donor liver transplantation cases of steatotic liver (n = 11)	Weight loss completely normalized liver function and lipid metabolism.	[165]
**Donor-recipient matching**	Steatotic liver graft	Liver transplant recipients of different liver grafts (steatotic, n = 2,048; non-steatotic, n = 69,394)	Donor-recipient matching could lower the mortality and risk of graft loss.	[51]
**Donor-recipient matching**	Steatotic liver graft	Liver transplantation cases (n = 77, 338)	The combination of low-fat livers and normal-BMI recipients had the lowest mortality.	[49]
**Donor-recipient matching**	DCD liver graft	Liver transplantation cases of different donors (DCD, n = 5,506; DBD, n = 107,685)	Prioritizing DCD organs for patients with lower MELD scores could optimize transplant outcomes.	[98]
**Machine perfusion**	Steatotic liver graft	Rats with severe hepatic macrosteatosis (≥60%)	Hypothermic oxygenated perfusion (HOPE) decreased reperfusion injury and fibrosis.	[71]
**Machine perfusion**	Steatotic liver graft	New Zealand White rabbits (n = 24)	Machine perfusion reduced tolerance to ischemia-reperfusion injury in steatotic livers.	[73]
**Machine perfusion**	DCD liver graft	Liver transplantation recipients (DBD, n = 447; cDCD with NRP, n = 144)	No difference in graft and patient survival between these two groups.	[102]
**Machine perfusion**	DCD liver graft	Liver transplantation recipients (DBD, n = 200; cDCD with NRP, n = 100)	NRP cDCD LT can achieve similar results to DBD LT.	[103]
**Machine perfusion**	DCD liver graft	Liver transplant recipients of different liver grafts (machine-perfused, n = 78; static cold storage, n = 78)	HOPE lowered the risk of non-anastomotic biliary strictures in DCD livers than conventional storage.	[104]
**Reduction of ischemic time**	DCD liver graft	Liver transplantation cases of DCD liver grafts (n = 1,567)	An hour of CIT was associated with a 6% increase in the relative rate of graft failure.	[106]
**Reduction of ischemic time**	DCD liver graft	Adult liver transplantation recipients of DCD liver grafts (n = 1,153)	Prolonged CIT could negatively affect the outcomes of DCD LT.	[107]
**Prebiotic heparin administration**	DCD liver graft	Adult liver transplantation recipients of DCD liver grafts (n = 3,754)	Administering heparin before the withdrawal of life-sustaining therapy could reduce the incidence of primary liver graft nonfunction.	[108]

#### 4.3.1 Donor-recipient matching

Given that the utilization of DCD transplantation is acknowledged as a notable risk factor for potential transplant failure, the guiding principles behind accepting DCD organs are aimed to minimize the cumulative risk factors between the donor and recipient. Specifically, for DCD liver recipients, it is necessary to minimize the complexity of the surgery and establish a more resilient environment for marginal grafts. This requires careful selection of recipients who can withstand the challenges of early allograft dysfunction (EAD) [99]. For high-risk donors, they need to be matched with low-risk recipients to shorten the CIT [100]. The MELD score is also the gold standard for DCD LT matching. Prioritizing the allocation of DCD organs to patients with lower MELD scores than their DBD counterparts emerges as a critical approach to optimizing both DCD utilization and ensuing outcomes [98]. By avoiding ineffective donor-recipient pairings, the utilization rate of DCD LT and the success rate of transplantation can be improved.

#### 4.3.2 Machine perfusion

Clinical liver machine perfusion systems for transplantation encompass various techniques such as NRP of the donor liver and ex vivo liver perfusion, either at normothermic or hypothermic temperatures [101]. Viguera *et al.* concluded that cDCD after NRP was not associated with increased red blood cell transfusion volume and had no difference in graft and patient survival compared with DBD [102]. In contrast to cases of brain death, emerging evidence suggests that cDCD LT following NRP, when matched based on donor age, recipient end-stage liver disease score model and CIT, can achieve similar results to DBD LT. Remarkably, 1- and 3-year overall transplant survival rates even surpass those of DBD, without cases of primary nonfunction or ischemic biliary injury [103]. In other words, cDCD following NRP not only offers a safer alternative but can also rival the results achieved by DBD LT, improving the imbalance between organ donors and the growing demand.

NMP represents a mechanism through which marginal livers can recover from warm ischemic injury during procurement, by delivering oxygen and nutrients at physiological temperature [98]. In comparison to conventional static cold storage, HOPE has been effective in mitigating the risk of post-transplantation NAS from DCD liver [104]. Additionally, ex vivo machine perfusion at normothermic temperature shows benefits in terms of easing graft tissue injury damage, enhancing graft survival rates and diminishing the occurrence of IRI [105]. Despite the promising potential of machine perfusion technology in the realm of DCD LT, comprehensive investigations are imperative to evaluate the efficacy of this rapidly evolving field in improving transplant quality and outcomes.

#### 4.3.3 Ischemic time reduction

In comparison to DBD livers, DCD livers experience more IRI, coupled with a heightened susceptibility to ischemic biliary complications due to WIT that ensues post-withdrawal of life support [98]. Furthermore, prolonged CIT has emerged as a negative factor impacting the outcomes of DCD LT [106, 107]. Thus, a pivotal strategy for enhancing the utilization of DCD livers lies in the reduction of ischemia time to mitigate IRI.

#### 4.3.4 Heparin administration

The administration of heparin before death has exhibited the potential to optimize graft survival [105, 108]. By administering heparin before the cessation of life-sustaining therapy, the incidence of PNF following LT can be diminished [108]. However, similar to cDCD LT, there were ethical concerns surrounding this practice [109].

## 5. Split liver grafts

### 5.1 Definition

**Figure 3.**
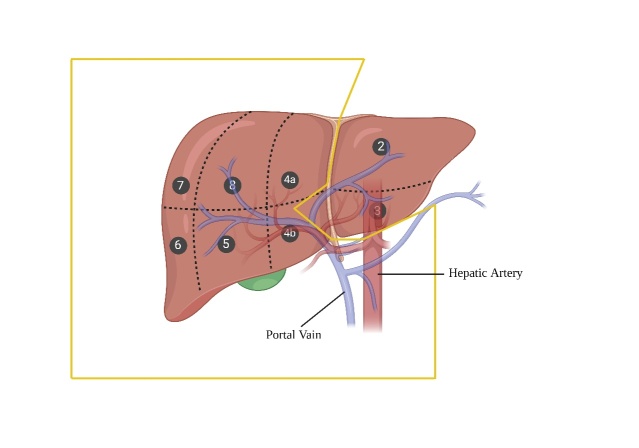
Split liver grafts for adult recipients.

Split LT (SLT) has emerged as a crucial strategy aimed at improving survival opportunities for critically ill young children with a high risk of mortality by ensuring access to appropriately sized deceased livers. The whole liver is meticulously divided into two transplantable segments, which is a technically challenging process. Typically, the allocation designates the left lateral segment (LLS) transplant (segments II and III) for pediatric or small adult recipients and an extended right lobe (ERL) transplant (segments I and IV-VIII) for adult recipients (Fig. 3, yellow line) [110]. This approach contributes, to a certain extent, to optimizing organ utilization, rendering LT a viable option for children and small adults [111]. Consequently, this initiative addresses the prevailing concern of organ scarcity. However, it is important to acknowledge that SLT demands a high degree of surgical expertise and may potentially elevate the perioperative risk, particularly in terms of biliary complications [112]. Moreover, there exists a possibility that a viable deceased organ might be transformed into two marginal transplants [111, 113]. Overall, SLT still should be given the priority of adapting if conditions permit.

Hence, a comprehensive evaluation of suitable donor organs for splitting and a careful selection process for prospective SLT recipients become paramount. Moreover, to enhance the number and safety of SLT procedures, it is imperative to adapt the logistical framework of the splitting procedure and fine-tune organ allocation policies [114]. In selected patients and experienced transplant centers, the outcome of SLT can approach those of whole organ LT under certain circumstances.

Ensuring secure biliary drainage across all segments assumes particular significance [105]. Notably, the cross-sectional area of the liver parenchyma is significantly larger compared to the conventional method. Furthermore, prioritizing in situ splitting is essential due to the absence of distinct anatomical markers such as the falciform ligament indicating the resection line. It is also necessary to carefully evaluate potential anatomical variations in blood vessels and bile ducts [106].

### 5.2 Usability evaluation

In recent years, propelled by the application of improved split techniques and optimal donor-recipient matching based on size, selection and timing, the transplant outcomes associated with SLT have exhibited commendable achievements, similar to whole LT (WLT) [116-119].

#### 5.2.1 Postoperative complications

Elevated susceptibility to biliary complications stands as a significant risk factor in SLT [112, 113, 120]. The use of microvascular arterial reconstruction should be considered as one might in the case of pediatric living donor liver transplantation. Moreover, the necessity for portal dissection and liver duct transection, coupled with the presence of a liver parenchymal section, increases the risk of biliary complications [121].

##### 5.2.1.1 Allograft rejection and graft loss

Nadalin *et al.* conducted a comprehensive analysis involving 316 adult patients who underwent SLT and 20,778 patients who received whole liver grafts, as reported in the UNOS database from 2002 to 2007. Their investigation unveiled no significant disparities in patient and graft survival rates between these groups at 6 and 12 months, regardless of the MELD score, particularly for patients with MELD scores >35 [122]. A retrospective analysis, focusing on pediatric recipients (<18 years) who had left lateral segmental grafts obtained from either living-related (LRD) or cadaveric in situ split-liver donors (SLD), was conducted. The results underscored that SLD grafts exhibited notably prolonged ischemia times and a heightened incidence of graft loss attributed to primary nonfunction and technical complications. It's worth noting that six of these graft losses within the SLD group were attributable to technical or immunologic causes [123]. Humar *et al.* also analyzed the survival rate in adult LT recipients and found that superior graft survival results were seen in living donor grafts versus split liver grafts of deceased donors [124].

##### 5.2.1.2 Vascular complications

Postoperative morbidity, including vascular complications, continues to pose a challenge for both pediatric and adult recipients undergoing SLT. Wan *et al.* conducted a meta-analysis to compare outcomes of right lobe SLT and WLT in adult patients. Their study found a significant difference in the incidence of overall vascular complications and HAT. Remarkably, the elevation in vascular complications was primarily observed following ex vivo SLT, rather than in situ SLT [125]. To comprehensively analyze the incidence and risk factors associated with vascular complications, a study involving 171 adult right lobe SLT procedures and 1412 WLT procedures were conducted. There was a greater overall incidence of vascular complications in the SLT than WLT group, but no difference in HAT [126]. Similarly, Oswari *et al.* investigated the survival and complication rates between split and reduced-size cadaveric grafts. However, their study did not discern a significant difference in the incidence of vascular complications between the two groups [127].

##### 5.2.1.3 Biliary complications

Bile leaks typically arise either from the exposed raw surface of the split liver or due to disruptions in the surgical biliary anastomosis [128]. In a study conducted by Wojcicki *et al.*, an analysis of 70 recipients revealed that 16 experienced biliary complications. Among these, the most prevalent biliary complication was a parenchymal radical leak from the transected liver surface in 8 patients. Additionally, anastomotic leaks were identified in 4 patients, while strictures were encountered in 3 patients [129]. Despite these challenges, the outcomes of extended right/left lateral splitting have shown remarkable long-term success. Notably, only a small percentage of cases require surgical revisions, with 10 percent in adult and 28 percent in pediatric patients [130].

##### 5.2.1.4 Recurrent liver diseases

Nowadays, there is limited research dedicated to recurrent liver disease and split liver grafts. Humar *et al.* [131] found that in 51 HCV-positive recipients who underwent LT (32 whole-liver (WL) transplants, 12 living donor (LD) transplants and 7 deceased donor (DD) split transplants), the incidence of histologic recurrence for these groups were 81%, 50% and 86% respectively. Within the DD split group, 2 recipients died, one from HCV recurrence with liver failure, the other from HCC recurrence and distant metastases. However, not all LT centers have identified this association and some investigations have reported comparable HCV recurrence rates between LD and DD split recipients [132, 133].

#### 5.2.2 Patient and graft survival

In a comparative context, utilizing complete right/left hepatic lobes for SLT in two adult recipients presents greater clinical challenges, both in terms of surgical technique and potential postoperative complications. Currently, most SLT outcomes remain uncertain, leading many transplant centers to adopt stringent donor selection criteria, typically favoring young, non-obese and hemodynamically stable donors. Similarly, recipient selection criteria, especially those necessitating urgent or re-transplantation, are rarely extended [120]. Research findings demonstrate that the 5-year survival rate of adult LT recipients (63% - 69%) tends to be lower than that of whole LT [134-137]. Nevertheless, recent reports have indicated a trend toward comparable outcomes among SLT, LDLT and whole LT [138-140].

The success of SLT in adult recipients has fueled an interest in broadening recipient selection criteria [120]. Chul *et al.* conducted a single-center study in Korea, comparing 86 adult patients who received ERGs with 303 adult patients who received WLGs. Their findings revealed that when patient criteria were restricted to a donor-recipient weight ratio >1.0, SLT exhibited similar overall survival rates to the WLG group, regardless of the MELD score [141]. This suggests that with adequate weight matching, SLT could potentially be suitable for patients with high MELD scores, thus justifying the expansion of recipient selection criteria. With further experiences and improved surgical techniques, the prospects for boosting the performance of SLT in adult recipients appear promising and may eventually overcome the current obstacles associated with the procedure.

### 5.3 Expanding the liver donor pool (Table 3)

#### 5.3.1 Donor-recipient matching

Precise donor and recipient selection, coupled with algorithmic matching, holds significant relevance for broadening the pool of liver donors, particularly in cases of marginal LT. The donor evaluation process should prioritize hemodynamic stability, optimal liver function, minimal hepatic steatosis and an age range of 15 to 55 years [142]. Recipient selection models incorporate multiple factors such as the UNOS score, MELD score and waiting time [142]. Within this allocation framework, LTs are typically prioritized for recipients exhibiting the most critical medical condition, as indicated by the highest UNOS status or MELD score.

#### 5.3.2 Machine perfusion

Normothermic machine perfusion combines the advantages of ex situ and in situ splitting techniques in liver splitting, which may reduce CIT and improve transplant quality [143]. Furthermore, when compared to static ex situ splitting, low temperature oxygenated perfusion of split liver shortens static cold preservation time, prolongs total ex situ preservation time and reduces neutrophil infiltration after reperfusion [144]. These findings suggest that establishing large-scale trials on machine perfusion in split LT could increase the utilization rate of split livers and improve their quality.

#### 5.3.3 Vein reconstruction

During the SLT process, the donor's liver is partitioned into an extended right graft and a left lateral segment, causing segment IV to undergo ischemia. To ensure optimal graft functionality and prevent potential complications, Wang *et al.* conducted a study involving portal vein reconstruction for segment IV in 14 patients. The study assessed variables such as surgical duration, intraoperative blood loss, liver function and postoperative complications [145]. Analysis between patients who underwent vascular reconstruction and those who did not reveal notable findings. The group that underwent segment IV portal vein reconstruction exhibited a substantial reduction in the ischemic area of segment IV, with no significant difference observed in surgical time among the two groups. Nevertheless, there was a significant difference in ALT on postoperative day 1 and albumin on day 6. Therefore, segment IV portal vein reconstruction during SLT surgery may reduce graft ischemia and promote its recovery.

#### 5.3.4 Cholangiography

During the phase of organ recovery, the donor surgeon should conduct a thorough examination of the liver to evaluate the size, vascular anatomy, and parenchymal consistency of the left and right lobes. Bile duct imaging evaluation of bile duct distribution is also necessary to determine the cut point of the left and right hepatic ducts [112]. The foremost challenge liver-splitting procedure lies in effectively identifying the shared major vessels and bile ducts, necessitating advancements in bile duct imaging techniques. Currently, the strategic division of major vessels and bile ducts occurs at an optimal point to facilitate streamlined reconstruction of vascular inflow and outflow, as well as bile duct drainage during graft implantation. This approach curbs the risk of complications stemming from intricate multiple anastomoses [142].

**Table 3:** Methods to expanding the liver donor pool in elderly liver and split liver transplantation.

Method	Type of liver graft	Research Subject	Outcomes	Ref.
**Donor-recipient matching**	Elderly liver graft	Liver transplantation cases of elderly liver grafts (n = 212)	Graft survival significantly decreased in patients with a high DR-MELD score.	[14]
**Donor-recipient matching**	Elderly liver graft	Liver transplantation recipients of different liver grafts (n = 38,891)	Donor-recipient matching significantly improved the outcomes of elderly liver grafts.	[35]
**Donor-recipient matching**	Elderly liver graft	Liver transplantation cases (n = 177)	Livers from octogenarian donors are acceptable for liver transplantation with donor-recipient matching.	[37]
**Donor-recipient matching**	Elderly liver graft	Liver transplantation cases of different liver grafts (donors≥80 yr, n = 33; others, n = 744)	Evaluation and selection can enable the use of liver grafts from donors aged over 80 years in LT.	[38]
**Vein reconstruction**	Split liver graft	Liver transplantation recipients of split liver grafts (with vein reconstruction, n = 14; without vascular reconstruction, n = 8)	IV segment portal vein reconstruction significantly decreased the ischemic area of the IV segment.	[145]
**In situ splitting**	Split liver graft	Adult liver transplantation recipients of split liver grafts (n = 100)	Graft weight was strongly associated with the initial success of split liver transplantation (SPLT).	[138]
**In situ splitting**	Split liver graft	Adult liver transplantation recipients of split liver grafts (n = 42)	Graft-recipient weight ratio was better more than 1% to avoid early patient death SPLT.	[140]
**In situ splitting**	Split liver graft	Liver transplantation cases of cadaveric right-sided grafts (n = 38)	The retransplantation rate was higher in right grafts than in extended right grafts.	[147]
**Machine perfusion**	Elderly liver graft	Liver transplantation cases of elderly liver grafts (n =14,796)	Reduction in CIT could significantly improve transplant outcomes.	[30]
**Machine perfusion**	Elderly liver graft	Adult liver transplantation recipients of different liver grafts (n = 71,926)	The only donor risk factor for old LT was a CIT exceeding 8 hours.	[40]
**Machine perfusion**	Elderly liver graft	Liver transplantation cases of different liver grafts (elderly, n = 2,162; non-elderly, n = 6,179)	Machine perfusion can improve transplant outcomes.	[41]
Machine perfusion	Split liver graft	Liver transplantations with split liver (HOPE, n = 17; static, n = 24)	HOPE-Split grafts prolonged total ex vivo preservation and reduced neutrophil infiltration on reperfusion biopsies.	[144]

#### 5.3.5 Splitting techniques

Depending on the donor's hemodynamic stability or the preference of the surgeon, the division of liver parenchyma can be executed through either in situ or ex situ methods [140, 146]. For hemodynamically stable donors, experienced LDLT transplant centers prefer in situ splitting [138, 140, 147] because in situ techniques reduce CIT and may improve graft quality compared to ex situ techniques [142]. The cut surface of the partial graft can be sealed with fibrin glue, which may help to reduce post-reperfusion bleeding [148].

#### 5.3.6 Improved split liver sharing policy

Following SLT in adult recipients, the allocation of the remaining half graft presents a logistical challenge within SLT, frequently linked with less favorable transplant results. Currently, there exists no standardized allocation system for the sharing of half liver grafts from SLT within the transplant community. Enhancing the policies for organ allocation and sharing is imperative to facilitate the broader integration of SLT into adult recipients [142]. Ethical and moral considerations regarding the splitting of donor livers also warrant careful consideration [111, 149].

## 6. Hepatitis virus-positive liver grafts

### 6.1 Definition

The surge in opioid drug usage has led to a surge in hepatitis virus-positive donors over the last decade, especially hepatitis B virus (HBV)/HCV-positive donors [150-152]. The advent of direct-acting antivirals (DAA) for hepatitis virus [153] has opened the door for increased use of HBV/HCV-infected donor organs in LT. However, their discard rate remains nearly twice that of hepatitis virus-negative livers [154]. The integration of virus/nucleic acid testing (NAT) plays a pivotal role in verifying active HBV/HCV infection, as NAT detects viral RNA with excellent specificity and remarkably low false positive rates [155]. Further optimization of hepatitis virus-positive liver utilization is needed to improve the access of all LT recipients.

### 6.2 Usability evaluation

#### 6.2.1 Postoperative complications

##### 6.2.1.1 Allograft rejection and graft loss

Yu *et al.* evaluated the LT outcomes from hepatitis B surface antigen (HBsAg)-positive or negative donors, and the results showed that 31.1% of graft losses occurred in the HBsAg-positive donor group, compared with 23.0% in the HBsAg-negative donor group. Among them, 58.4% of graft losses occurred in patients with HCC [156]. In 2022, Snyder *et al.* conducted a review of existing literature on the treatment outcomes of HCV viremia in LT recipients who were HCV-negative (non-viremic) and received organs from HCV viremic donors. A total of 253 LT patients were evaluated for sustained virologic response, and 99.6% of patients achieved a cure with minimal drug adverse reactions. 23 patients experienced rejection, 12 died and 1 was lost [157]. Furthermore, another research compared the difference between HCV+ and HCV- grafts in HCV+ recipients, finding that donor hepatitis C status didn’t impact on graft or patient survival after LT for HCV+ recipients [158].

##### 6.2.1.2 Transmission of viral hepatitis

Muñoz *et al.* stated that the liver of a donor previously exposed to HBV may fail due to severe HBV reactivation in recipients [159]. To estimate the incidence of HCV transmission, Bari *et al.* prospectively followed 26 consecutive HCV antibody-negative (n = 25) or nucleic acid test (NAT)-negative (n = 1) transplant recipients who received a liver graft from donors who were HCV antibody-positive but serum NAT-negative. The results unveiled that HCV transmission occurred in 4 of these recipients, within a median follow-up period of 11 months [160]. A study found that among 34 HCV-negative patients who received organs from HCV-positive donors (20 viremic and 14 non-viremic), all 20 patients who received viremic transplants were confirmed to have HCV viremia within 3 days after LT [153].

#### 6.2.2 Patient and graft survival

In recent years, the accumulation of experience involving the use of DAA therapy and HBV/HCV viremic LT in HBV/HCV-negative recipients, has yielded promising results in terms of treatment tolerance and immediate outcomes [151, 160-162]. It was shown that in the HBsAg-positive group, the 1-, 3-, and 5-year patient and graft survival rates were lower than those of the HBsAg-negative group, while the difference was not significant [156]. Cotter *et al.* analyzed the long-term outcomes of LT from HCV-positive donors to HCV-positive recipients and compared them with those of HCV-negative recipients from HCV-negative and positive donors (D- / R-, D- / R+ and D+ / R+), using the US Scientific Registry of Transplant Recipients database from January 1, 2008, to June 30, 2018 [163]. The findings of this study underscored the overall enhancement in graft survival across all recipient groups in the DAA era, with the most remarkable strides witnessed among HCV-positive recipients. Significantly, the 3-year survival rate of HCV-positive patients who received HCV-positive donor livers aligned closely with that of their HCV-negative counterparts who were the recipients of HCV-positive donor livers.

### 6.3 Expanding the liver donor pool

Given the heightened mortality experienced by patients on transplantation waiting lists and the marked efficacy of antiviral therapies, the transplantation community should embrace the utilization of hepatitis virus-positive donor livers to create transplant opportunities for specific recipients without viral hepatitis. Particularly, adopting an approach where any liver becomes acceptable for transplantation when the MELD score is ≥20 could yield a substantial boost in life expectancy. This allocation strategy might also prove to be economically prudent [162]. Lu *et al.* proposed a new concept about the matching status of recipient hepatitis B core antigen and donor HBsAg, which showed excellent recurrence prediction ability in both training and validation cohorts, and significantly reduced the recurrence risk in the low-risk group compared to the high-risk group, with a 3-year survival rate of 94.9% [164]. By incorporating both non-viremic and viremic donor livers into the equation, the waiting times and mortality rates for potential HCV-negative recipients could be significantly diminished. However, it remains paramount to exercise judicious discretion when selecting both donors and recipients, maintaining vigilant oversight of potential complications and ensuring the timely commencement of antiviral treatment protocols upon transplantation. As we navigate this evolving landscape, it is imperative to underscore the need for extended follow-up through clinical trials to conclusively validate and reinforce these promising outcomes.

**Figure 4.**
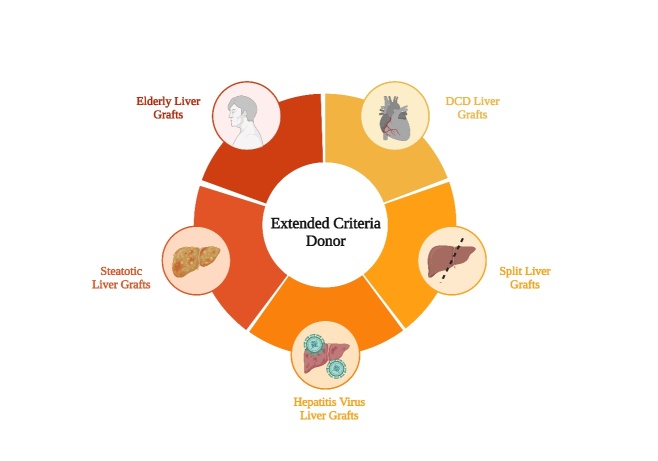
Extended criteria donor.

## 7. Conclusions

As LT techniques have become more widely adopted and the population of individuals with end-stage liver disease continues to grow, the critical issue of donor liver scarcity has gained prominence. This has led to the gradual application and importance of marginal donor livers. The careful assessment and strategic incorporation of these marginal donor livers offers a dual advantage: alleviating the donor shortage dilemma while concurrently optimizing liver utilization, thereby extending benefits to a larger patient cohort. Nowadays, numerous clinical studies have shown that adequate evaluation, matching of donors and organ preservation contribute to the application of marginal liver grafts. This is particularly evident in the rapid advancements of extracorporeal liver perfusion techniques, which have significantly augmented the quality of marginal donor livers. Consequently, the once elevated rate of discarded marginal livers has declined, while the prognosis of liver transplantation recipients has improved markedly. As a result, what were once considered "marginal donors" are progressively shifting into the realm of mainstream donor sources. Nonetheless, the potential ethical problems of many marginal donors cannot be ignored. Therefore, the next step for marginal donor livers should be to improve the evaluation system and matching allocation system for all types of liver sources, to promote the improvement of liver preservation techniques and translate promising new therapies into clinical practice (Fig. 4).
